# Sinulariolide Suppresses Cell Migration and Invasion by Inhibiting Matrix Metalloproteinase-2/-9 and Urokinase through the PI3K/AKT/mTOR Signaling Pathway in Human Bladder Cancer Cells

**DOI:** 10.3390/md15080238

**Published:** 2017-08-02

**Authors:** Te-Chih Cheng, Zhong-Hao Din, Jui-Hsin Su, Yu-Jen Wu, Chih-I Liu

**Affiliations:** 1 Department of Nursing, Mei-ho University, Pingtung 91202, Taiwan; x00003077@meiho.edu.tw; 2 Graduate Institute of Applied Healthy and Biotechnology, Mei-ho University, Pingtung 91202, Taiwan; a0981709760@gmail.com; 3 Taiwan Coral Research Center, National Museum of Marine Biology and Aquarium, Pingtung 94446, Taiwan; x2219@nmmba.gov.tw; 4 Department of Biological Technology, Mei-ho University, Pingtung 91202, Taiwan

**Keywords:** sinulariolide, human bladder cancer, migration, invasion, PI3K/AKT/mTOR signaling pathway

## Abstract

Sinulariolide is a natural product extracted from the cultured-type soft coral *Sinularia flexibilis*, and possesses bioactivity against the movement of several types of cancer cells. However, the molecular pathway behind its effects on human bladder cancer remain poorly understood. Using a human bladder cancer cell line as an in vitro model, this study investigated the underlying mechanism of sinulariolide against cell migration/invasion in TSGH-8301 cells. We found that sinulariolide inhibited TSGH-8301 cell migration/invasion, and the effect was concentration-dependent. Furthermore, the protein expressions of matrix metalloproteinases (MMPs) MMP-2 and MMP-9, as well as urokinase, were significantly decreased after 24-h sinulariolide treatment. Meanwhile, the increased expression of tissue inhibitors of metalloproteinases (TIMPs) TIMP-1 and TIMP-2 were in parallel with an increased concentration of sinulariolide. Finally, the expressions of several key phosphorylated proteins in the mTOR signaling pathway were also downregulated by sinulariolide treatment. Our results demonstrated that sinulariolide has significant effects against TSGH-8301 cell migration/invasion, and its effects were associated with decreased levels of MMP-2/-9 and urokinase expression, as well as increased TIMP-1/TIMP-2 expression. The inhibitory effects were mediated by reducing phosphorylation proteins of the PI3K, AKT, and mTOR signaling pathway. The findings suggested that sinulariolide is a good candidate for advanced investigation with the aim of developing a new drug for the treatment of human bladder cancer.

## 1. Introduction

Human bladder cancer is one of the most common cancers in the United States [1] and the ninth most common worldwide [2], accounting for approximately 5% of the mortality rate of all types of cancer in the US. It has been reported that bladder cancer causes more than 150,000 deaths worldwide, and has been estimated to cause 15,500 deaths per year in the United States alone [3,4]. The majority of human bladder cancers are diagnosed as urothelial carcinoma, known as transitional cell carcinoma (TCC), which is derived from the transitional epithelium [5]. TCCs are usually classified into non-invasive and invasive bladder cancer, or referred to as superficial and advanced bladder cancer, respectively. Non-invasive tumors remain within the transitional epithelium layer, whereas invasive tumors progress to grow deeper into the muscle layer of the bladder wall, and metastatic cells are likely to spread through the lymphatic system [6,7].

Urothelial carcinoma is not only one of the most highly prevalent cancers diagnosed in adults; the incidence rate in the elderly is also increasing, particularly in the aging female population [8]. Although the combined use of surgery, radiotherapy, and chemotherapy is the most common means by which to treat advanced bladder cancer, it is often not an ideal option for the treatment of metastatic bladder cancer, for which the therapeutic outcomes are often limited and unsatisfactory. Therefore, alternative therapeutic approaches such as immunotherapy and molecular targeted therapy through regulating signaling pathways of genomic and proteomic expressions are of potential use in the diagnosis and treatment of metastatic TCC [9]. Carcinoma metastasis represents malignancy, tumor progression, and cancer mortality. The migration and invasion of metastatic carcinoma cells have been found to lead to destruction of the extracellular matrix (ECM), and several signal transduction pathways are known to be involved in the processes.

Two proteolytic enzymes—matrix metalloproteinases (MMPs) MMP-2 and MMP-9—have been found to have extensively elevated levels in malignant tumors. Expressions of these enzymes are major characteristics of malignant invasion and metastasis of cancer cells, as these enzymes are able to function to degrade the ECM, and may promote the penetration of cancer cells into the basement membrane. MMP activation is facilitated by serine proteinases of the plasminogen/plasminogen activator system in a cascade reaction. Plasminogen proteins are cleaved by urokinase (also called urokinase-type plasminogen activator; uPA), and are converted to plasmin with serine proteinase activity to activate MMPs. Studies have indicated that urokinase is also involved in tumor cell proliferation, metastasis, and invasion, as well as angiogenesis. The activity of MMPs is blocked by endogenous protein regulators, known as tissue inhibitors of metalloproteinases (TIMPs). TIMPs are specific inhibitors of MMPs, and thus a correct balance between MMPs and TIMPs is crucial in normal tissue [10]. Imbalance between MMPs and their inhibitors leads to ECM degradation, which promotes the metastasis of cancer cells. Therefore, MMP-2/-9 and urokinase have important roles in the pathological processes of ECM degradation, carcinoma metastasis, and cell invasion. Inhibition of the protein expressions and enzyme activities of MMP-2/-9 and urokinase is considered to represent a potential therapeutic strategy to suppress cancer metastasis.

Sinulariolide is an active natural product that was originally isolated from cultured-type soft coral *Sinularia flexibilis*. Recently, it has been shown to possess activities against cancer cells—in particular inducing apoptosis in bladder cancer and melanoma cells [11,12]. Sinulariolide has also been found to suppress human hepatocellular carcinoma cell migration and invasion [13]. Therefore, in the present study, we explored the molecular mechanism of its inhibition effects on human bladder cancer cell migration and invasion. Overall, our results provided valuable information related to the use of sinulariolide in bladder cancer drug development.

## 2. Results

### 2.1. Sinulariolide Treatment and Cell Viability

**Figure 1:**
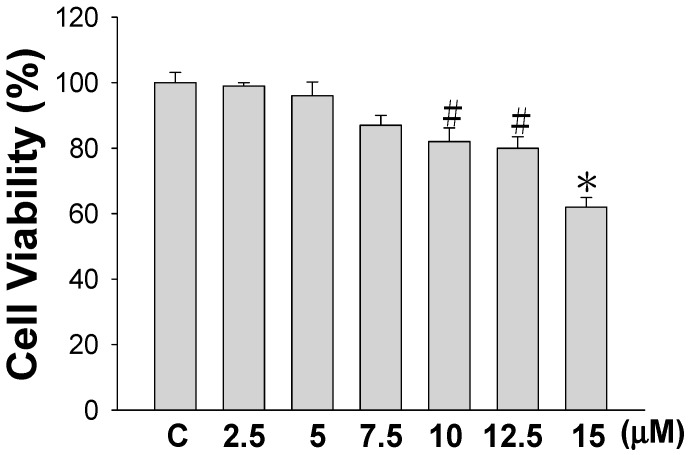
Cell Viability effect of sinulariolide on TSGH-8301 cells. TSGH-8301 cells were treated with different concentrations of sinulariolide or vehicle control for 24 h, and cell numbers in each group were measured using MTT assays. The results are expressed as a percentage of the control, and significant differences are denoted (^#^ *p* < 0.05, * *p* < 0.001).

Before studying how sinulariolide affects cell migration and invasion, we first examined the cytotoxicity of sinulariolide against human bladder cancer TSGH-8301 cells. Using a 3-(4,5-dimethylthiazol-2-yl)-2,5-diphenyltetrazolium bromide (MTT) assay, a significant decrease in TSGH-8301 cell viability was seen in cells treated with 10 μM or a higher concentration of sinulariolide, as shown in Figure 1. As a 20% reduction of the cell viability effect was seen in cells treated with a concentration of 10 μM, concentrations of 5, 7.5, and 10 μM were used in subsequent experiments in this study to ensure that the inhibitory effects of sinulariolide on TSGH-8301 cell migration and invasion were not caused by the cytotoxicity of the compound.

### 2.2. Sinulariolide Affects Cell Migration and Cell Invasion

**Figure 2:**
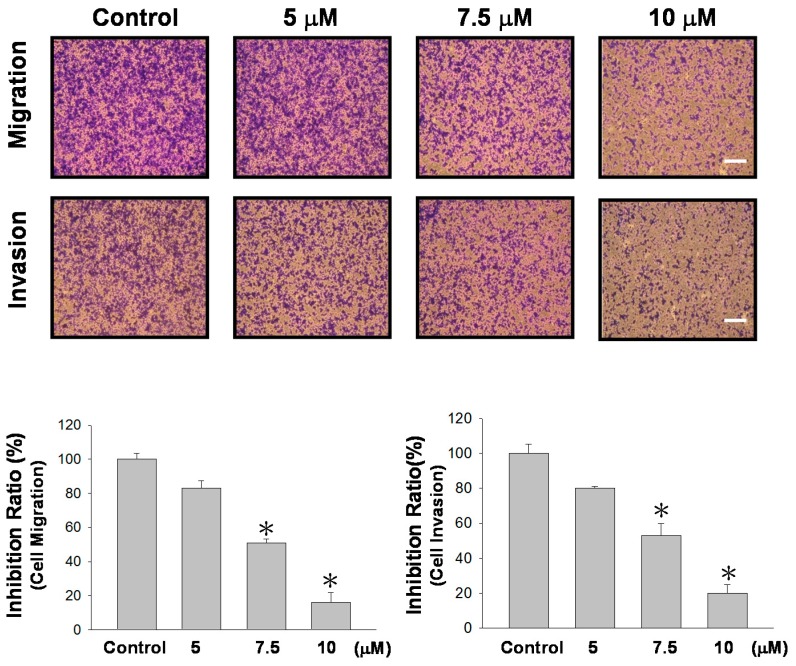
Sinulariolide inhibited TSGH-8301 cell migration and invasion. Sinulariolide inhibited TSGH-8301 cell migration and penetration through Transwell membranes. Control: cells treated with DMSO vehicle control (*n* = 3; three independent experiments, * *p* < 0.01).

We next examined the effects of sinulariolide on TSGH-8301 cells in terms of their migration and invasion through membrane inserts. The results indicated that sinulariolide inhibited TSGH-8301 cell migration and invasion at a concentration of 7.5 μM or higher, and greater cell migration/invasion were observed with increased sinulariolide concentrations (Figure 2). A concentration-dependent effect was seen, and concentrations of 7.5 and 10 μM had significant inhibition effects (*p* < 0.05).

### 2.3. Sinulariolide Regulates the Protein Expressions of MMP-2/-9, Urokinase, and TIMP-1/-2

**Figure 3:**
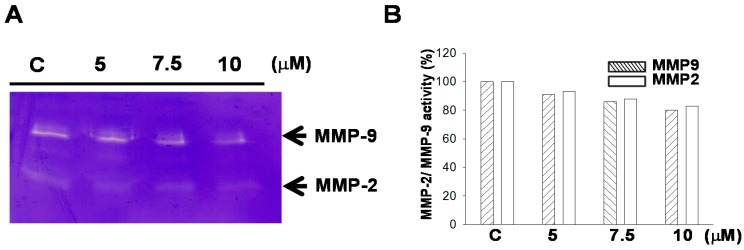
Sinulariolide suppressed matrix metalloproteinase (MMP)-2 and MMP-9 activities. TSGH-8301 cells were incubated with different concentrations of sinulariolide (5, 7.5, and 10 μM) for 24 h. (**A**) In the end of incubation period, the culture media were collected, and gel zymography with gelatin was used to measure MMP-2 and MMP-9 activities. (**B**) Quantification of MMP-2 and MMP-9 using Image J 1.47 software (National Institutes of Health, Bethesda, MD, USA).

**Figure 4:**
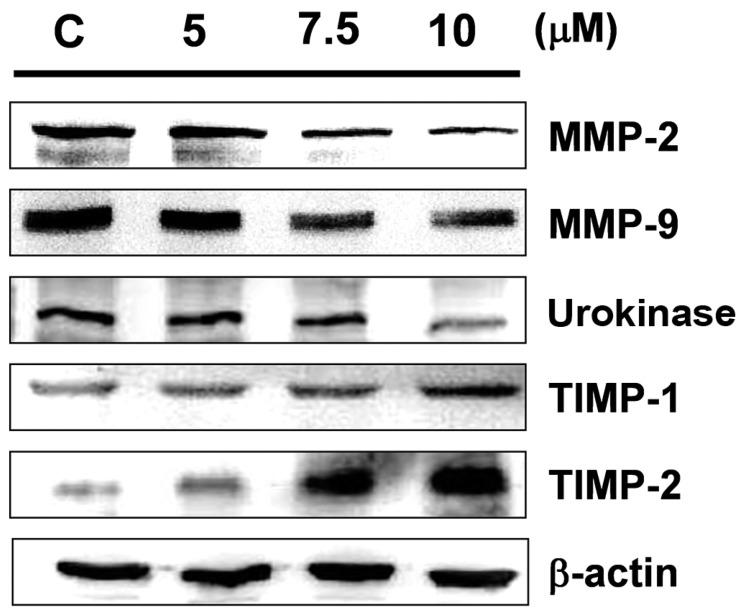
Sinulariolide suppressed MMP-2/-9 and urokinase protein expressions, and augmented tissue inhibitor of metalloproteinases (TIMP)-1/-2 protein expressions. Total cell lysates from TSGH-8301 cells treated with sinulariolide were analyzed in terms of their expression levels of MMP-2, MMP-9, urokinase, and TIMP-1/-2 by Western blotting. C: cells treated with DMSO vehicle only. β-actin was used as the protein loading control.

MMP-2/-9 are extracellular enzymes that promote ECM degradation and promote the movement of tumor cells [14,15]. Therefore, the expression levels of MMP-2 and MMP-9 can be used as indicators of cell motion and invasive activity, as well as the potential to induce angiogenesis in certain cell types. Gel zymography with gelatin was used in this study to detect secreted MMP-9 and MMP-2 activity. Figure 3 shows that sinulariolide treatment reduced the enzyme activity of MMP-2 and MMP-9. We further used Western blotting analysis to quantify the expressions of MMP-2, MMP-9, and related proteins. The results indicated that sinulariolide inhibited the protein expression levels of MMP-2/-9 and urokinase, although the levels of TIMP-1/-2 were increased (Figure 4).

### 2.4. Sinulariolide Influences the mTOR Signaling Pathway

**Figure 5:**
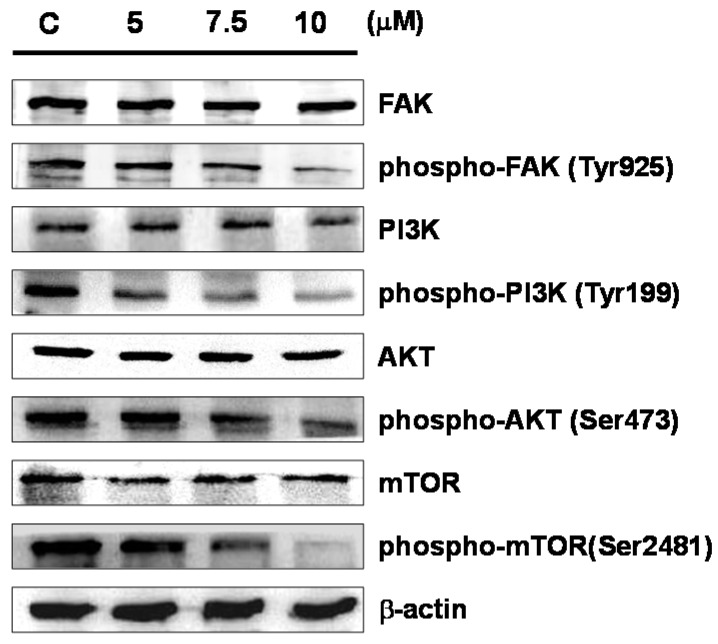
Sinulariolide regulated the expressions of key molecules of the mTOR signaling pathway in TSGH-8301 cells. Western blotting data demonstrated altered profiles of the expressions of focal adhesion kinase (FAK), phosphorylated FAK (phospho-FAK), phosphoinositide 3-kinase (PI3K), phospho-PI3K, AKT, phospho-AKT, mTOR and phospho-mTOR in TSGH-8301 cells treated with sinulariolide. C: cells treated with DMSO vehicle only. β-actin was used as the protein loading control.

We next investigated whether the effects of sinulariolide on cell migration and invasion could be attributed to the possible involvement of the FAK/PI3K/AKT/mTOR signaling pathway (mTOR signaling pathway in short hereafter). Western blotting analysis demonstrated that cells treated with higher concentrations of sinulariolide had lower levels of phosphorylated focal adhesion kinase (FAK), phosphoinositide 3-kinases (PI3K), AKT, and mTOR, while the total protein levels of these molecules were unaffected following sinulariolide treatment (Figure 5).

### 2.5. Inhibition of PI3K Reduced the Cell Migration and MMP-2/MMP-9 and Urokinase Protein Expression

**Figure 6:**
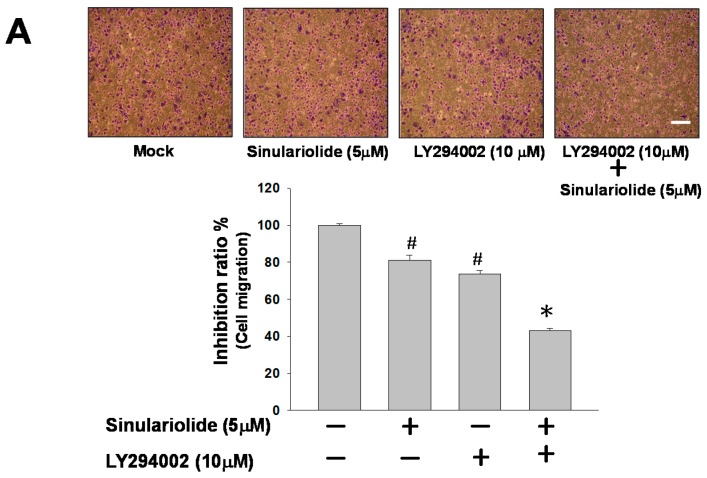

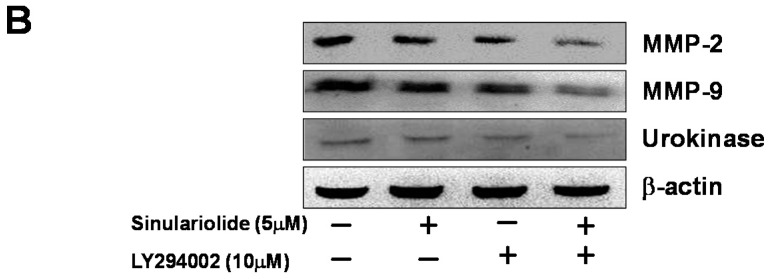
Inhibition of PI3K reduces the cell migration and MMP-2/MMP-9 and urokinase protein expression in TSGH-8301 cells. (**A**) Sinulariolide and LY294002 inhibited TSGH-8301 cell migration and penetration through Transwell membranes. (**B**) Western blotting showed the protein expression profiles of MMP-2/MMP-9 and urokinase in TSGH-8301 cells treated with sinulariolide (5 μM) and LY294002 (10 μM). β-actin was used as the protein loading control.

To further examine the association between sinulariolide with the aforementioned PI3K/AKT pathways, LY292400—a PI3K inhibitor—was used to elucidate the effects on cell migration inhibited by sinulariolide through the PI3K/AKT pathway. The results indicated that the cell migration of the sinulariolide-treated TSGH-8301 cells reduced from 80% to 41% after treatment with LY292400 (10 mM) (Figure 6A). Moreover, the expression levels of MMP-2, MMP-9, and urokinase showed a significant reduction in sinulariolide-treated TSGH-8301 cells with the addition of LY292400 (Figure 6B). Therefore, we proposed that the cell migration of TSGH-8301 cells should be suppressed by sinulariolide through the PI3K/AKT pathway.

### 2.6. Sinulariolide Inhibits the Expressions of Cell Migration- and Invasion-Related Proteins

**Figure 7:**
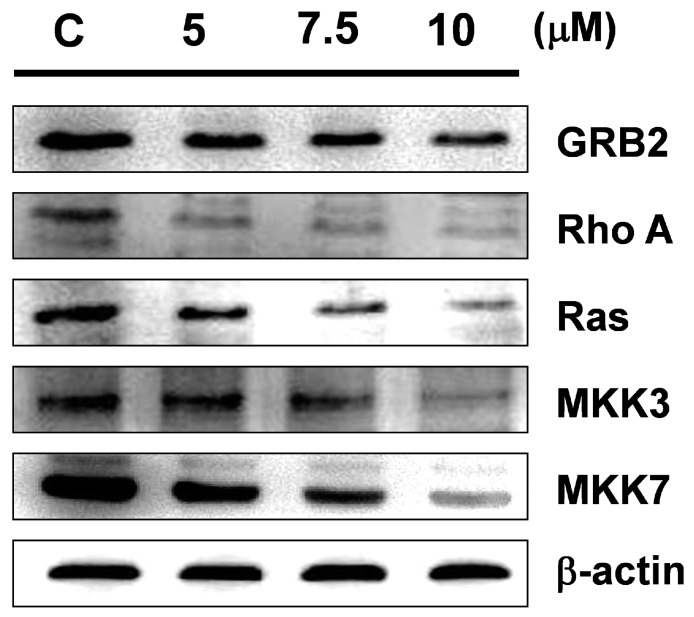
Sinulariolide treatment reduced the amounts of proteins associated with cell migration and invasion in TSGH-8301 cells. Western blotting showed the protein expression profiles of growth factor receptor-bound protein 2 (GRB2), RhoA, Ras, mitogen-activated protein kinase kinase 3 (MKK3), and MKK7 in TSGH-8301 cells treated with various concentrations of sinulariolide. C: cells treated with DMSO vehicle only. β-actin was used as the protein loading control.

In order to identify the impacts of sinulariolide on cell migration- and invasion-related proteins, we analyzed the protein expressions of Ras, RhoA, growth factor receptor-bound protein 2 (GRB2), mitogen-activated protein kinase kinase 7 (MKK7), and MKK3 in cells after sinulariolide treatment by Western blotting. The results indicated that sinulariolide inhibited the expressions of all these tested proteins, of which the expressions of GRB2, Ras, RhoA, MKK3, and MKK7 were inhibited by sinulariolide in a concentration-dependent manner (Figure 7).

## 3. Discussion

The metastasis of cancer cells involves cell migration and invasion, and the mechanisms of cell migration and invasion include the binding of cell surface receptors to their ligands and initiation of downstream molecules in the signaling mechanisms. This further results in the activation of relevant target signaling pathways, and leads to increased reorganization of the cytoskeleton [16]. In the current therapeutic approach, inhibition of the mechanism associated with tumor cell migration/invasion is the key to controlling cancer metastasis [17]. Many of the active ingredients isolated from corals have been shown to possess properties that prevent cancer cell proliferation and metastasis [12,18,19,20,21,22,23].

The development and application of new anti-cancer drugs are considered to be extremely important. In previous studies, sinulariolide has been found to inhibit growth and induce programmed cell death in hepatocellular carcinoma cells, and may prevent metastasis of liver cancer [13]. In addition, a study also reported that sinulariolide promoted apoptosis in bladder cancer cells through the processes of mitochondrial inactivation and activation of p38AMPK [11]; however, no study has investigated whether it can inhibit cell migration and invasion in bladder cancer cells. In the present study, we used Transwell migration and invasion assays to assess the effects of sinulariolide on bladder cancer cells. Our results indicated that sinulariolide inhibited cell migration and invasion in TSGH-8301 bladder cancer cells in a dose-dependent manner (Figure 2).

At concentrations that resulted in significant inhibition of cell migration and invasion (at a concentration of 10 μM), no significant cytotoxicity was observed (Figure 1). The results indicated that the inhibition effects on cell migration and invasion were not due to toxicity of sinulariolide to the cells. The secretion of MMP-2/-9 and urokinase by tumors to degrade and cleave the ECM could be a strategy by which tumors gain the capacity for invasion and metastasis [24]. In particular, in tumors in the early stages, the prevention of overexpression of MMPs and the reduction of the expressions of integrins and ECM proteins have been suggested to represent a useful strategy by which to control tumor growth. A balance of TIMPs and MMPs can control the local activities of MMPs in tissues to avoid the degradation of ECM proteins; however, a disturbed balance of TIMPs and MMPs is known to occur in malignant tumors, which leads to cancer cell migration and invasion. Study has shown that TIMP-1/-2 and MMP-9 all play crucial roles in the tumor cell growth and invasion of hepatocellular carcinomas [25,26]. 11-*epi*-Sinulariolide acetate was originally isolated from cultured-type soft coral *Sinularia flexibilis*, and has been reported to exert anti-invasion and anti-migration effects on hepatoma HA22T cells [27]. We also found that sinulariolide treatment decreased the protein expression levels of MMP-2/-9 and increased TIMP-1/-2 protein expressions in TSGH-8301 cells. As shown in Figure 4, the results suggested that the decreases in TSGH-8301 cell migration and invasion after sinulariolide treatment were regulated by MMP-2/-9 and TIMP-1/-2 interaction.

Studies of the molecular mechanism of the development of malignancy have shown that FAK contributes to cancer-cell motility and invasive activity by controlling the interaction between the ECM microenvironment and the tumor cells [28,29]. FAK phosphorylates substrates as a scaffold, and focal adhesion induces signal transduction pathways that promote MMPs secretion, leading to degradation of ECM substrates and increased tumor cell adhesion on ECM substrates [30]. In addition, apart from being relevant to cell growth, the mTOR signaling pathway is also known to be involved in the survival and differentiation of cells, as well as their invasive potential [31]. Furthermore, many studies have demonstrated that mTOR signaling contributes to the regulation of MMP-2/-9 activities [32,33,34]. In this study, we found that sinulariolide treatment only inhibited the immunoreactivities of phosphorylated proteins in the PI3K/AKT/mTOR pathway (including FAK, PI3K, AKT, and mTOR), and did not influence the level of total protein expression in the cells (Figure 5). In order to ensure that these signaling factors are responsible for the sinulariolide-induced inhibitory effects on cell migration, we blocked PI3K/AKT signaling in the presence of sinulariolide and evaluated cell migration in TSGH-8301 cells. Our results demonstrated that the specific PI3K inhibitor LY294002 significantly suppressed the cell migration, and markedly inhibited the MMP-2/MMP-9 and urokinase proteins expression (Figure 6). The findings support that sinulariolide may inhibit the signaling pathways related to cell migration, which is also inhibited by PI3K/AKT signaling.

In an invasive breast cancer cell line, GRB2 was found to regulate GTPases activation, and may also trigger ATF4 and ATF6 [35]. Rho GTPases control the actin cytoskeleton and promote cancer cell invasion [36,37]. Another study demonstrated that treatment that suppressed RhoA signaling reduced the migration and invasive activity of human ovarian cancer cells [38,39]. In our study, we found that sinulariolide treatment reduced the protein expression levels of GRB2, RhoA, Ras, MKK7, and MKK3 (Figure 7). Therefore, we inferred that sinulariolide induced reduction of MMP-2, MMP-9, and urokinase protein expressions in TSGH-8301 cells, and cell migration and invasion activities were regulated by inhibition of the mTOR signaling pathway.

In conclusion, sinulariolide inhibited TSGH-8301 cell migration and invasion, which involved decreases in the expressions of MMP-2/-9 and urokinase, and increased TIMP-1/-2 protein expressions. Our study of the signaling pathways involved in the inhibitory effects of sinulariolide showed that the mechanism is mediated by reductions in phosphorylated FAK, PI3K, AKT, and mTOR proteins. Our findings suggested that sinulariolide is a great candidate for further development as a new drug for the treatment of bladder cancer.

## 4. Material and Methods

### 4.1. Materials and Antibodies

Sinulariolide was prepared from the extract of cultured-type soft coral *Sinularia flexibilis*, following the previously published protocol [40,41]. The compound was dissolved in dimethyl sulfoxide (DMSO). Rabbit anti-human β-actin antibody, 3-(4,5-dimethylthiazol-2-yl)-2,5-diphenyltetrazolium bromide (MTT), LY294002, and other general chemicals were obtained from Sigma-Aldrich Corporation (St Louis, MO, USA). Goat anti-rabbit IgG with horseradish peroxidase (HRP) conjugate was purchased from EMD Millipore (Billerica, MA, USA). Chemiluminescent substrate for western HRP development was obtained from Pierce (Rockford, IL, USA). Rabbit antibodies against human MKK3, MKK7, GRB2, FAK, mTOR, phosphorylated-mTOR, and RhoA were obtained from Epitomics Inc. (Burlingame, CA, USA). Rabbit antibodies against human TIMP-1 and TIMP-2 were purchased from ProteinTech Group Inc. (Rosemont, IL, USA). Rabbit antibodies against human MMP-2, MMP-9, urokinase, PI3K, and phosphorylated-PI3K were obtained from Cell Signaling Technology Inc. (Danvers, MA, USA).

### 4.2. Cell Culture and MTT Assay

Human bladder cancer TSGH-8301 cells were purchased from the Taiwan Food Industry Research and Development Institute (Hsinchu, Taiwan). The cells were treated with various concentrations of sinulariolide (0, 2.5, 5, 7.5, 10, 12.5, 15 μM) and incubated for 24 h before harvesting for further analyses. The viability of TSGH-8301 cells after sinulariolide treatment was assessed using MTT assays, as described previously [12]. TSGH-8301 cells were plated on 24-well polystyrene plates at a seeding cell density of 1 × 10^5^ cells per well. After 24 h of incubation with different concentrations of sinulariolide, the cells were processed following the MTT procedure. The plates were then analyzed using a microplate ELISA reader (Bio-Rad; Hercules, CA, USA) at the manufacturer’s suggested setting. Samples for the MTT assay were analyzed in triplicate and were repeated at least three times for all the experiments.

### 4.3. Cell Migration and Invasion Assays

The methods published by Neoh and coworkers [11] were used for the cell migration assay. Briefly, TSGH-8301 cells in serum-free media were plated into an uncoated Boyden chamber (Neuro Probe; Cabin John, MD, USA) at 5 × 10^4^ cells per well. TSGH-8301 cells with or without sinulariolide treatment were cultured in a 37 °C CO_2_ incubator for 24 h to allow cells to migrate through the membrane. The invasion assay was performed as previously described by Yeh and colleagues [42]. Briefly, Transwell inserts with 8 µm-pore-size polycarbonate membrane filters with a Matrigel coating (10 µL 0.5 mg/mL each well; BD Biosciences; Franklin Lakes, NJ, USA) were used, and TSGH-8301 cells were seeded onto the coated membrane of the upper chamber. Regular cell culture medium containing serum was placed in the bottom chamber. At the end of incubation period, the cells invaded through the Matrigel-coated membrane to the lower chamber, and were then fixed with ice-cold methanol. After staining with Giemsa solution (concentration = 5%; Merck; Darmstadt, Germany), the cells were counted under a light microscope.

### 4.4. Determination of MMP-2/-9 Activities by Zymography

Zymography assays with gelatin were used to determine MMP-2/-9 enzyme activities in the culture medium from TSGH-8301 cells following sinulariolide treatment. The experiment was performed per the method described previously [43]. Briefly, TSGH-8301 cells were treated with sinulariolide at various concentrations (5, 7.5, 10 μM) for 24 h. After concentrating the medium samples using a vacuum concentrator‎, MMP-2/-9 secreted from the cells were loaded onto non-reducing SDS-PAGE (10%) containing 0.2% gelatin for separation. After washing three times in 100 mM NaCl/50 mM Tris-HCl (pH = 7.5) buffer containing 2.5%, the enzymes in the gels were incubated at 37 °C for 24 h in 200 mM NaCl/50 mM Tris-HCl buffer (pH = 7.5) containing 1 mM CaCl_2_, 0.02% NaN_3_, 1 µM ZnCl_2_, and 2% Triton-X 100 for activation. The gels were then stained with standard protocol (with Coomassie blue R-250 dye), further de-stained, and then the activities of MMP-2/-9 were quantified using ImageJ software (NIH; Bethesda, MD, USA).

### 4.5. Proteins Estimated and Western Blot Assay

TSGH-8301 cells (3 × 10^5^ cells) cultured in 10 cm dish were incubated in FBS-DMEM media with different concentrations of sinulariolide (0, 5, 7.5, and 10 µM) for 24 h and then lysed with Cell Extraction Buffer (BioSource International, Camarillo, CA, USA). The protein contents were determined using Bradford protein assay (Bio-Rad). Sinulariolide-treated samples and DMSO-treated control samples (total proteins = 25 μg) were separated by SDS-PAGE (12.5%), followed by transferring onto PVDF membrane (electric current = 400 mA, for 1.6 h). The membrane containing transferred proteins was blocked in phosphate-buffered saline (PBS) buffer containing 5% low-fat milk powder to eliminate nonspecific binding. After incubation with primary antibodies at 4 °C overnight, the transferred membrane was incubated with secondary antibodies (dilution = 1/5000 in blocking solution) for 2 h at 4 °C. Chemiluminescence substrate solution (Pierce Biotechnology; Rockford, IL, USA) was used for visualization of the protein signals on the membrane.

### 4.6. Statistical Analysis

Data from the MTT assay, cell migration assay from the Boyden chamber, and invasion assay from Transwell inserts derived from three independent experiments were collected for analyses. Analysis of variance (ANOVA) and the Tukey–Kramer test were used. The results were then plotted using Graphpad Instat software (San Diego, CA, USA).

## References

[1] National Cancer Institute Cancer Stat Facts: Bladder Cancer. https://seer.cancer.gov/statfacts/html/urinb.html.

[2] American Cancer Society (2015). American Cancer Society. Global Cancer Facts & Figures.

[3] Jemal A., Bray F., Center M.M., Ferlay J., Ward E., Forman D. (2011). Global cancer statistics. CA Cancer J. Clin..

[4] Siegel R., Ma J., Zou Z., Jemal A. (2014). Cancer statistics, 2014. CA Cancer J. Clin..

[5] Toll A.D., Epstein J.I. (2012). Invasive low-grade papillary urothelial carcinoma: A clinicopathologic analysis of 41 cases. Am. J. Surg. Pathol..

[6] Pasin E., Josephson D.Y., Mitra A.P., Cote R.J., Stein J.P. (2008). Superficial bladder cancer: An update on etiology, molecular development, classification, and natural history. Rev. Urol..

[7] Tsukamoto T., Kitamura H., Takahashi A., Masumori N. (2004). Treatment of invasive bladder cancer: Lessons from the past and perspective for the future. Jpn. J. Clin. Oncol..

[8] Shi B., Zhang K., Zhang J., Chen J., Zhang N., Xu Z. (2008). Relationship between patient age and superficial transitional cell carcinoma characteristics. Urology.

[9] Ikeda S., Hansel D.E., Kurzrock R. (2015). Beyond conventional chemotherapy: Emerging molecular targeted and immunotherapy strategies in urothelial carcinoma. Cancer Treat. Rev..

[10] Jackson H.W., Defamie V., Waterhouse P., Khokha R. (2017). TIMPs: Versatile extracellular regulators in cancer. Nat. Rev. Cancer.

[11] Neoh C.A., Wang R.Y., Din Z.H., Su J.H., Chen Y.K., Tsai F.J., Weng S.H., Wu Y.J. (2012). Induction of apoptosis by sinulariolide from soft coral through mitochondrial-related and p38mapk pathways on human bladder carcinoma cells. Mar. Drugs.

[12] Li H.H., Su J.H., Chiu C.C., Lin J.J., Yang Z.Y., Hwang W.I., Chen Y.K., Lo Y.H., Wu Y.J. (2013). Proteomic investigation of the sinulariolide-treated melanoma cells A375: Effects on the cell apoptosis through mitochondrial-related pathway and activation of caspase cascade. Mar. Drugs.

[13] Wu Y.J., Neoh C.A., Tsao C.Y., Su J.H., Li H.H. (2015). Sinulariolide Suppresses Human Hepatocellular Carcinoma Cell Migration and Invasion by Inhibiting Matrix Metalloproteinase-2/-9 through mapks and PI3K/Akt Signaling Pathways. Int. J. Mol. Sci..

[14] Yang S.F., Chen M.K., Hsieh Y.S., Yang J.S., Zavras A.I., Hsieh Y.H., Su S.C., Kao T.Y., Chen P.N., Chu S.C. (2010). Antimetastatic effects of Terminalia catappa L. on oral cancer via a down-regulation of metastasis-associated proteases. Food Chem. Toxicol..

[15] Yeh C.B., Hsieh M.J., Hsieh Y.H., Chien M.H., Chiou H.L., Yang S.F. (2012). Antimetastatic effects of norcantharidin on hepatocellular carcinoma by transcriptional inhibition of MMP-9 through modulation of NF-kb activity. PLoS ONE.

[16] Anand-Apte B., Zetter B. (1997). Signaling mechanisms in growth factor-stimulated cell motility. Stem Cells.

[17] Fenteany G., Zhu S. (2003). Small-molecule inhibitors of actin dynamics and cell motility. Curr. Top. Med. Chem..

[18] Liu C.I., Chen C.C., Chen J.C., Su J.H., Huang H.H., Chen J.Y., Wu Y.J. (2011). Proteomic analysis of anti-tumor effects of 11-dehydrosinulariolide on CAL-27 cells. Mar. Drugs.

[19] Hassan H.M., Khanfar M.A., Elnagar A.Y., Mohammed R., Shaala L.A., Youssef D.T., Hifnawy M.S., El Sayed K.A. (2010). Pachycladins A-E, prostate cancer invasion and migration inhibitory Eunicellin-based diterpenoids from the red sea soft coral Cladiella pachyclados. J. Nat. Prod..

[20] Kamel H.N., Ferreira D., Garcia-Fernandez L.F., Slattery M. (2007). Cytotoxic diterpenoids from the hybrid soft coral Sinularia maxima x Sinularia polydactyla. J. Nat. Prod..

[21] Poza J.J., Fernandez R., Reyes F., Rodriguez J., Jimenez C. (2008). Isolation, biological significance, synthesis, and cytotoxic evaluation of new natural parathiosteroids A-C and analogues from the soft coral Paragorgia sp.. J. Org. Chem..

[22] Andrianasolo E.H., Haramaty L., White E., Lutz R., Falkowski P. (2014). Mode of action of diterpene and characterization of related metabolites from the soft coral, Xenia elongata. Mar. Drugs.

[23] Arepalli S.K., Sridhar V., Rao J.V., Kennady P.K., Venkateswarlu Y. (2009). Furano-sesquiterpene from soft coral, Sinularia kavarittiensis: Induces apoptosis via the mitochondrial-mediated caspase-dependent pathway in THP-1, leukemia cell line. Apoptosis.

[24] Weng C.J., Chau C.F., Hsieh Y.S., Yang S.F., Yen G.C. (2008). Lucidenic acid inhibits PMA-induced invasion of human hepatoma cells through inactivating MAPK/ERK signal transduction pathway and reducing binding activities of NF-kappab and AP-1. Carcinogenesis.

[25] Nakatsukasa H., Ashida K., Higashi T., Ohguchi S., Tsuboi S., Hino N., Nouso K., Urabe Y., Kinugasa N., Yoshida K. (1996). Cellular distribution of transcripts for tissue inhibitor of metalloproteinases 1 and 2 in human hepatocellular carcinomas. Hepatology.

[26] Ordonez R., Carbajo-Pescador S., Prieto-Dominguez N., Garcia-Palomo A., Gonzalez-Gallego J., Mauriz J.L. (2014). Inhibition of matrix metalloproteinase-9 and nuclear factor kappa B contribute to melatonin prevention of motility and invasiveness in hepg2 liver cancer cells. J. Pineal Res..

[27] Lin J.J., Su J.H., Tsai C.C., Chen Y.J., Liao M.H., Wu Y.J. (2014). 11-epi-Sinulariolide acetate reduces cell migration and invasion of human hepatocellular carcinoma by reducing the activation of ERK1/2, p38mapk and FAK/PI3K/AKT/mtor signaling pathways. Mar. Drugs.

[28] Mclean G.W., Carragher N.O., Avizienyte E., Evans J., Brunton V.G., Frame M.C. (2005). The role of focal-adhesion kinase in cancer—a new therapeutic opportunity. Nat. Rev. Cancer.

[29] Parsons J.T. (2003). Focal adhesion kinase: The first ten years. J. Cell Sci..

[30] Canel M., Secades P., Garzon-Arango M., Allonca E., Suarez C., Serrels A., Frame M., Brunton V., Chiara M.D. (2008). Involvement of focal adhesion kinase in cellular invasion of head and neck squamous cell carcinomas via regulation of MMP-2 expression. Br. J. Cancer.

[31] Tian T., Nan K.J., Guo H., Wang W.J., Ruan Z.P., Wang S.H., Liang X., Lu C.X. (2010). PTEN inhibits the migration and invasion of hepg2 cells by coordinately decreasing MMP expression via the PI3K/Akt pathway. Oncol. Rep..

[32] Brouxhon S.M., Kyrkanides S., Teng X., Athar M., Ghazizadeh S., Simon M., O’ Banion M.K., Ma L. (2014). Soluble E-cadherin: A critical oncogene modulating receptor tyrosine kinases, MAPK and PI3K/Akt/mtor signaling. Oncogene.

[33] Yang N., Hui L., Wang Y., Yang H., Jiang X. (2014). SOX2 promotes the migration and invasion of laryngeal cancer cells by induction of MMP-2 via the PI3K/Akt/mtor pathway. Oncol. Rep..

[34] Chan K.C., Ho H.H., Huang C.N., Lin M.C., Chen H.M., Wang C.J. (2009). Mulberry leaf extract inhibits vascular smooth muscle cell migration involving a block of small gtpase and Akt/NF-kappab signals. J. Agric. Food Chem..

[35] Haines E., Saucier C., Claing A. (2014). The adaptor proteins p66Shc and Grb2 regulate the activation of the gtpases ARF1 and ARF6 in invasive breast cancer cells. J. Biol. Chem..

[36] Etienne-Manneville S., Hall A. (2002). Rho gtpases in cell biology. Nature.

[37] Ellenbroek S.I., Collard J.G. (2007). Rho gtpases: Functions and association with cancer. Clin. Exp. Metastasis.

[38] Hwang H., Kim E.K., Park J., Suh P.G., Cho Y.K. (2014). Rhoa and Rac1 play independent roles in lysophosphatidic acid-induced ovarian cancer chemotaxis. Integr. Biol..

[39] Marjoram R.J., Lessey E.C., Burridge K. (2014). Regulation of rhoa activity by adhesion molecules and mechanotransduction. Curr. Mol. Med..

[40] Hsieh P.W., Chang F.R., McPhail A.T., Lee K.H., Wu Y.C. (2003). New cembranolide analogues from the formosan soft coral Sinularia flexibilis and their cytotoxicity. Nat. Prod. Res..

[41] Tsai T.-C., Chen H.-Y., Sheu J.-H., Chiang M.Y., Wen Z.-H., Dai C.-F., Su J.-H. (2015). Structural Elucidation and Structure–Anti-inflammatory Activity Relationships of Cembranoids from Cultured Soft Corals Sinularia sandensis and Sinularia flexibilis. J. Agric. Food Chem..

[42] Yeh C.B., Hsieh M.J., Hsieh Y.S., Chien M.H., Lin P.Y., Chiou H.L., Yang S.F. (2012). Terminalia catappa Exerts Antimetastatic Effects on Hepatocellular Carcinoma through Transcriptional Inhibition of Matrix Metalloproteinase-9 by Modulating NF-kappab and AP-1 Activity. Evid. Based Complement. Altern. Med..

[43] Chen K., Zhang S., Ji Y., Li J., An P., Ren H., Liang R., Yang J., Li Z. (2013). Baicalein inhibits the invasion and metastatic capabilities of hepatocellular carcinoma cells via down-regulation of the ERK pathway. PLoS ONE.

